# Oral and ocular manifestations in a patient with coronavirus disease-2019: Clinical presentation and management

**DOI:** 10.1590/0037-8682-0699-2021

**Published:** 2022-04-08

**Authors:** Alparslan Dilsiz, Emine Parlak, SemaNur Sevinc Gül

**Affiliations:** 1 Atatürk University, Faculty of Dentistry, Department of Periodontology, Erzurum, Turkey.; 2 Atatürk University, Faculty of Medicine, Department of Infectious Diseases and Clinical Microbiology, Erzurum, Turkey.; 3 Atatürk University, Faculty of Dentistry, Department of Periodontology, Erzurum, Turkey.

**FIGURE 1:**
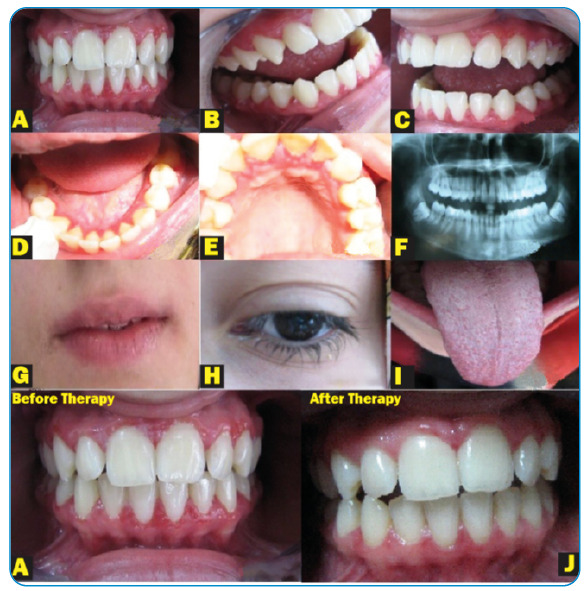
**(A)** Initial view. Note reddish/whitish lesions and peeling of the maxillary and mandibular attached gingiva. (Before therapy); **(B)** initial clinical intraoral view of the associated right region; **(C)** initial facial view of the associated left region; **(D)** clinical lingual view of the anterior mandible. Note diffuse erythema present on the gingiva; **(E)** clinical palatal view of the anterior maxillary. Note diffuse erythema and ulcer present on the gingiva; **(F)** panoramic radiograph view; **(G)** initial view. Note cheilitis present on the lips; **(H)** initial clinical aspect of the patient’s eye **(conjunctivitis)**; **(I)** clinical aspect of the patient’s tongue **(strawberry tongue)**; **(J)** view of the case 14 days after all therapy. The regular appearance was established **(after therapy)**.

An 18-year-old girl who complained of painful oral ulceration and severe burning sensation on the gingiva was admitted to the Periodontology Department (Figure 1A-F). SARS-CoV-2 RT-PCR was positive 4 days previously.

Erythema, blisters, peeling, whitish fibrin-coated ulcers, and desquamation of the affected oral mucosa during examination. The condition was diagnosed as desquamative gingivitis due to SARS-CoV-2 infection. Other findings included gingival bleeding, edema, severe tenderness, aphthous-like lesions on the buccal mucosa, white exudate-coated strawberry tongue, mouth dryness, halitosis, loss of taste, and smell. In addition, slight cheilitis, peeling of the lips, and bilateral conjunctivitis were observed. Ophthalmologic examination revealed mild bilateral conjunctivitis. Slight scleritis was observed in the lower eyelids (Figure 1G-I).

The affected areas were gently cleansed with hydrogen peroxide, followed by mechanical debridement, and a 10% povidone-iodine solution was applied. The patient was instructed to perform oral irrigation with a 0.5% fresh sodium hypochlorite solution. The ophthalmologist prescribed 0.15% ganciclovir gel and polyvinyl alcohol+povidone drops. Two weeks later, the SARS-CoV-2 RT-PCR results were negative. The affected areas healed completely without complications (Figure 1J).

Coronavirus disease-19 (COVID-19) has some oral manifestations such as ageusia, desquamative gingivitis, and oral ulcerations^1^ ^,^ ^2^; however, whether it is specific to the disease remains controversial. Additionally, ocular manifestations are usually uncommon, but very indicative of COVID-19^3^. Desquamative gingivitis and conjunctivitis in patients with SARS-CoV-2 infection were treated with ophthalmic and periodontal therapy. New information is needed to describe the signs and symptoms of COVID-19 and therapies and take protective measures.
